# Association between the expression of vascular endothelial growth factors and metabolic syndrome or its components: a systematic review and meta-analysis

**DOI:** 10.1186/s13098-018-0363-0

**Published:** 2018-08-03

**Authors:** Mohammad Ishraq Zafar, Kerry Mills, Xiaofeng Ye, Brette Blakely, Jie Min, Wen Kong, Nan Zhang, Luoning Gou, Anita Regmi, Sheng Qing Hu, Juan Zheng, Lu-Lu Chen

**Affiliations:** 1 Department of Endocrinology, Union Hospital, Tongji Medical College, Huazhong University of Science and Technology, Wuhan, People’s Republic of China; 2 Health Research Institute, University of Canberra, Canberra, Australia; 3 Centre for Healthcare Resilience and Implementation Science (CHRIS), Australian Institute of Health Innovation, Macquarie University, Sydney, NSW Australia

**Keywords:** Hypertriglyceridemia, Hyperglycemia, Body mass index, Obesity, Hypertension, Diabetes, Type 2 diabetes, Type 1 diabetes, Growth factors, Insulin resistance, Impaired glucose tolerance, Metabolic syndrome, Diabetes mellitus, Hypercholesterolemia

## Abstract

**Background:**

Several studies have linked vascular endothelial growth factors (VEGFs) with metabolic syndrome or its components. However, there has been no systematic appraisal of the findings of these studies to date. The current systematic review and meta-analysis was conducted to explore this association.

**Methods:**

PubMed, EMBASE, the Cochrane library, and clinical trials registries were used to retrieve peer-reviewed clinical studies that had evaluated the association of VEGFs with metabolic syndrome or its components without applying language and date restrictions. The final search was performed on 29 September 2017.

**Results:**

We included 32 studies in this systematic review and meta-analysis, of which 16 studies (19 study arms) were included in the meta-analysis and remaining studies were qualitatively assessed. Overall, VEGF-A, VEGF-B and VEGF-C were strongly associated with metabolic syndrome or its components. The components of metabolic syndrome varied in their association. Obesity was not correlated with increased VEGF-A expression (p = 0.12), whereas VEGF-B and VEGF-C expression was significantly higher in those with obesity. In contrast, hyperglycemia in type 1 diabetes was strongly associated with increased VEGF-A levels (p < 0.00001), as was type 2 diabetes (p = 0.0006). The studies included in the qualitative analysis similarly showed an increase in VEGF family expression in people with metabolic syndrome, and with its components.

**Conclusion:**

The increased concentrations of vascular endothelial growth factors are variably associated with metabolic syndrome or its components. Each VEGF protein has a unique set of associations with the disease state.

## Background

According to the World Health Organization (WHO) definition, the metabolic syndrome (MetS) comprises insulin resistance (IR) (defined as impaired fasting glucose, impaired glucose tolerance or type 2 diabetes mellitus (T2DM)), associated with at least two of the following: obesity (waist-to-hip ratio > 0.90 in men or > 0.85 in women, or BMI > 30 kg/m^2^), dyslipidemia (TGs ≥ 150 mg/dL and/or HDL-C < 35 mg/dL in men or < 39 mg/dL in women) and hypertension (BP ≥ 140/90 mmHg) [1]. The MetS directly increases the risk of atherosclerotic cardiovascular disease and all-cause mortality, and in the current obesity era, it demands novel therapeutic strategies to protect and treat the affected population [1]. A perturbed crosstalk between adipocytes and endothelial cells (ECs) has been found to play a key role in the pathogenesis of obesity and consequent metabolic disturbances [2, 3]. Communication between adipocytes and ECs mostly takes place through the vascular endothelial growth factors (VEGFs) and their receptors (VEGFR1, VEGFR2, and VEGFR3), thus the role of these factors in metabolic disturbances has been the focus of scientific attention in recent years [3, 4]. Clarifying the association between VEGFs and parameters of the metabolic syndrome holds both therapeutic and preventive potential, and as such, requires a systematic analysis of the existing data.

Clinical research on four VEGF proteins (VEGF-A, VEGF-B, VEGF-C, and VEGF-D) in the context of the metabolic syndrome have yielded diverse results. VEGF-A-induced angiogenesis has been found to diminish metabolic complications caused by a high-fat diet and the metabolic syndrome. Accordingly, there is evidence that increased circulating and adipose tissue levels of VEGF-A in obesity significantly decrease in patients with a dramatic weight loss [5–7]. The metabolic role of VEGF-B, on the other hand, seems to be more complex than that of VEGF-A [8]. While some authors report increased circulating and adipose tissue VEGF-B levels [9] in obese individuals, others disagree [10]. A further study in mice implicates increased VEGF-B expression in reducing metabolic complications [11]. The metabolic roles of VEGF-C, VEGF-D, and a VEGF discovered in human placenta called placental growth factor (PIGF) have not been studied as extensively as those of VEGF-A and VEGF-B, but the evidence suggests that circulating VEGF-C is significantly increased in obese as compared to lean individuals, whereas both circulating and adipose tissue levels of VEGF-D seem to be significantly lower in obese patients as compared to lean individuals, showing a positive correlation with the degree of insulin resistance [9, 10]. Furthermore, blockade of VEGF-C and VEGF-D in mice reduces adipose tissue inflammation and improves insulin sensitivity [12].

The ability of the VEGF superfamily to increase perfusion and therefore improve insulin delivery in adipose tissues could be crucial for the treatment of insulin resistance. Furthermore, these proteins may play a role in preventing lipotoxicity and improving insulin signaling through the regulation of FA uptake. It is therefore important to explore the expression of these proteins in such patients [8]. Since there has been no systematic appraisal of the findings regarding the metabolic role of VEGFs to date, we aimed to systematically review and quantify all the available data on the expression of VEGF-A, VEGF-B, VEGF-C, VEGF-D and PIFG in adults, adolescents and children with metabolic syndrome or its components.

## Methods

The systematic review and meta-analysis was carried out in accordance with the PRISMA guidelines [13], and the review was registered in the PROSPERO International Prospective Register of Systematic Reviews (CRD42017077685).

### Review question (PICOTS)

The question for our study was: In children, adolescents and adults, are people with metabolic syndrome, or the components of metabolic syndrome, compared with controls, associated with higher concentrations of VEGF family members in cross-sectional or cohort studies?

### Data sources and search strategies

A comprehensive systematic search was carried out using PubMed, the Cochrane Library, EMBASE and international clinical trials registries without time or language restrictions. The final search was conducted on 29 September 2017. The search strategy as used for PubMed was; (“vascular endothelial growth factor” OR “VEGF-A” OR “VEGF-B” OR “VEGF-C” OR “VEGF-D” OR “PIGF”) AND (“metabolic syndrome” OR “diabetes” OR “T2DM” OR “obesity” OR “overweight” OR “hypertension” OR “hyperglycemia” OR “high blood sugar” OR “hypertriglyceridemia” OR “low-density lipoprotein” OR low-HDL” OR “high-density lipoprotein” OR “insulin resistance” OR “insulin resistance syndrome”) AND (“fasting blood glucose” OR “FBG” OR “fasting blood insulin” OR “FBI” OR HOMA-IR” OR “postprandial insulin” OR “postprandial glucose” OR “body mass index” OR “BMI” OR “body fat composition” OR “fasting metabolic rate” OR “HbA1c” OR “glycated haemoglobin” OR “body weight”).

The search was modified for used in EMBASE, the Cochrane Library and the clinical trials registries.

### Inclusion criteria

Citations were included if they met the following criteria: the study was in children, adolescents, and or adults; the participants in at least one cohort had metabolic syndrome or at least one component of the metabolic syndrome; the expression of VEGF-A or a member of the VEGF superfamily was reported, and a comparison was drawn between the individuals with or without metabolic syndrome or its components. The studies were excluded if they did not meet the inclusion criteria. Articles without full text, conference abstracts, comments, reviews, and studies based on animal models or cell lines were excluded.

### Study selection and quality assessment

The citations and full-text articles were assessed for inclusion or exclusion independently by two authors using the double-blind coding assignment methodology within EPPI-Reviewer 4 [14]. The quality of the studies was determined using the National Institute of Health Quality Assessment Tool for Observational Cohort and Cross-Sectional Studies [15]. Two authors assessed and rated the quality of the included studies. Any disagreements regarding the article selection or quality assessment were resolved by consensus, or by reference to a third party.

### Data extraction

The data extraction was performed by two authors independently. Each author was assigned half of the total included studies, and two authors individually checked the extracted data. The data included: study characteristics (disease state, country, study type, population, age, gender, and funding source), VEGF family member measured, and VEGF expression levels. If the data were only available in figures, the data were extracted using WebPlotDigitizer [16]. Standard errors were converted to standard deviations using the equation: $SD = SEM \times \sqrt N$, where N is the number in the study arm.

### Statistical analysis

Extracted data were analyzed using Review Manager 5.3 [17]. As the data were reported in different formats, we calculated standardized mean differences (SMD) with 95% confidence intervals (CI). To convert SMDs to mean differences (MD), we multiplied the SMD by the median standard deviation of studies reporting the outcome of choice using the same format. To avoid double-counting, the population size of study arms that were used twice were divided by two.

The meta-analysis was done using a random-effects, inverse variance model, as the differences in effect sizes were expected to be modified by variations in populations. Heterogeneity was reported as τ^2^, χ^2^, and I^2^. A funnel plot was created to identify potential publication bias in the included studies. All statistical results were considered to be significant if the p-value was < 0.05.

The potential for publication bias was tested by measuring asymmetry in the funnel plot as determined by Egger et al. [18] using the regtest.rma function in the metaphor package (Version 2.0-0) [19].

### A priori subgroup analysis/sensitivity analysis

We planned a priori subgroup analyses based on: concomitant medication, funding source, age of participants, co-morbidities of participants and gender of participants.

## Results

### Study characteristics

**Fig. 1:**
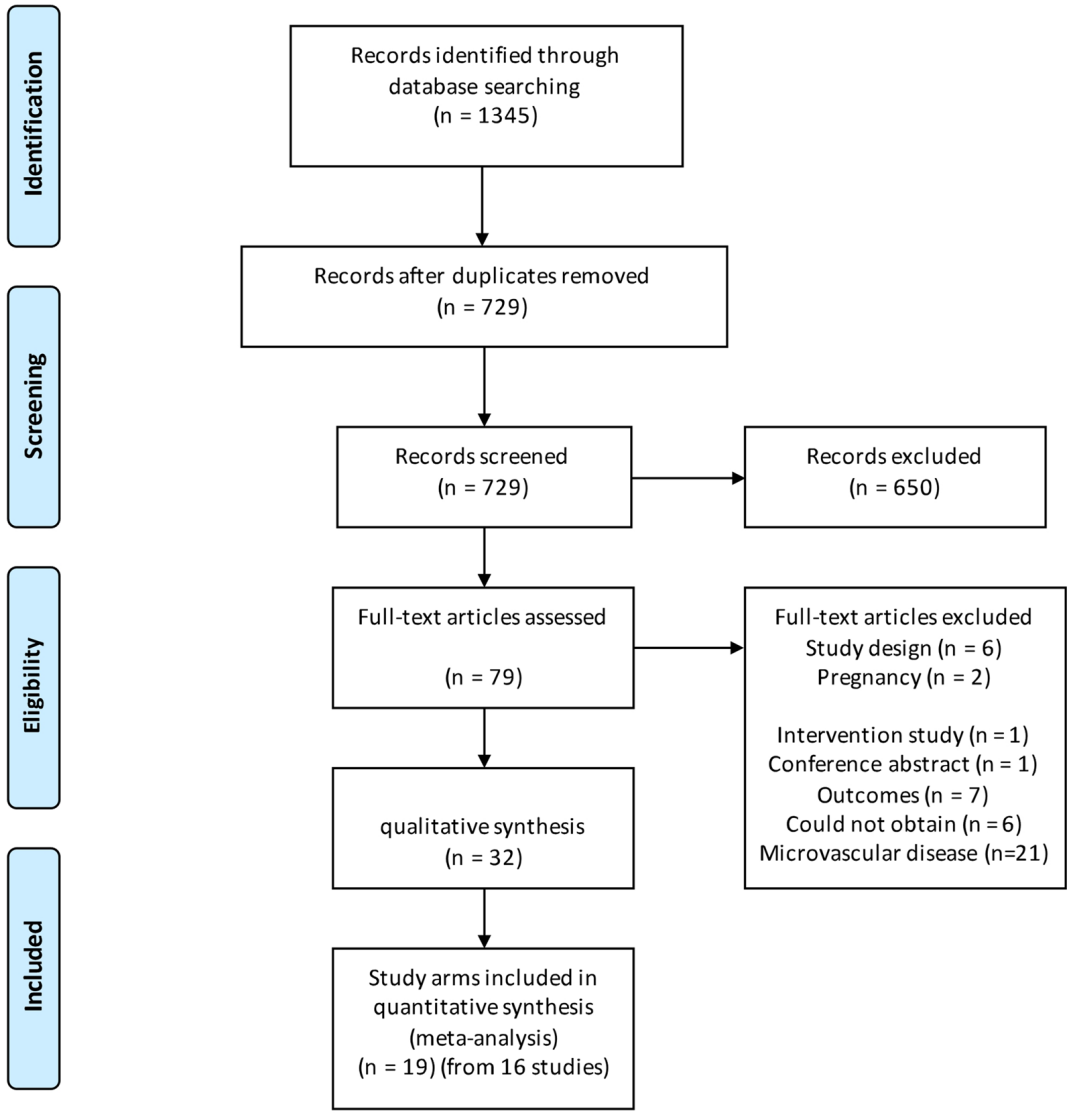
PRISMA flow diagram

In total, 1345 citations were uploaded into EPPI-Reviewer 4 [14] (Fig. 1). After removal of duplicates, 729 abstracts were subjected to inclusion as stated above. After coding at the title/abstract level, 79 full texts were obtained. Of these, 32 studies including 36 study arms were included in the final analysis (Table 1) [9, 20–50]. Of these, 19 study arms from 16 studies were included in the meta-analysis.

**Table 1:** Characteristics of included studies

Study ID	Disease state	Control type	VEGF protein(s) measured	Place measured	Country	Funding	N disease group	N control	Average age	Gender	Metabolic syndrome?
Doupis 2011/1 [50]	Diabetes without obesity	Healthy	VEGF-A	Serum	USA	NIH	76	40	55	Both	No
Doupis 2011/2 [50]	Diabetes with obesity	Healthy	VEGF-A	Serum	USA	NIH	105	37	54	Both	Yes
Du 2016 [20]	Diabetes	Healthy	VEGF-A	Serum	China	Government grant	25	20	57.3	Both	No
Erman 2016 [21]	Metabolic syndrome	Healthy	VEGF-A	Serum	Turkey	No information	45	17	53.67	No info	Yes
Gomez-Ambrosi 2010 [9]	Obese	Healthy	VEGF-A, VEGF-B, VEGF-C, VEGF-D	Serum	Spain	Institutional grant	24	15	34.4	Both	No
Guo 2014 [22]	Diabetic hypertension	Healthy	VEGF-A	Serum	China	Government grant	18	26	60.5	Both	No
Hanefeld 2016 [23]	T2DM	Healthy	VEGF-A	Serum, plasma	Germany	Industry grant	302	99	67.36	Both	No
Jain 2013 [24]	T2DM	Healthy	VEGF-A	Serum	Switzerland	No information	19	19	52	Both	No
Jesmin 2013 [25]	Metabolic syndrome	Healthy	VEGF-A	Plasma	Japan	Government grant	451	1322	45.92	Female	Yes
Kakizawa 2004 [26]	Diabetes	Healthy	VEGF-A	Plasma	Japan	University grant	45	54	50.9	Both	No
Kubisz 2010 [27]	T2DM	Healthy	VEGF-A	Serum	Slovakia	University grant	42	42	61.8	Both	No
Lim 2004/1 [28]	Diabetes CVD+	Healthy	VEGF-A	Plasma	UK	No information	38	34	68	Both	Yes
Lim 2004/2 [28]	Diabetes CVD-	Healthy	VEGF-A	Plasma	UK	No information	56	34	68	Both	No
Litvinova 2014 [29]	Metabolic syndrome	Healthy	VEGF-A	Serum	Russia	Government grant	23	10	50.7	Female	Yes
Loebig 2010 [30]	Obese	Healthy	VEGF-A	Plasma	Germany	Institutional grant	15	15	27.8	Male	No
MacEneaney 2010 [31]	Obese sedentary	Healthy sedentary	VEGF-A	Supernatant	America	Government/charity grant	42	25	56	Both	No
Mahdy 2011 [32]	T2DM	Healthy	VEGF-A	Serum	Egypt	No information	10	10	58	Both	No
Marek 2010 [33]	T1DM	Healthy	VEGF-A	Serum	Poland	Government grant	60	30	16.31	Both	No
Mirhafez 2015 [34]	Metabolic syndrome	Healthy	VEGF-A	Serum	Iran	Government grant	155	148	53.91	Both	Yes
Mirhafez 2016 [35]	High triglycerides	Healthy	VEGF-A	Serum	Iran	University grant	95	260	50.5	Both	Yes
Mysliwiec 2008 [36]	T1DM	Healthy	VEGF-A	Serum	Poland	No information	163	85	12.58	Both	No
Nandy 2010 [37]	T2DM	Healthy	VEGF-A	Plasma	America	Government/university grant	8	11	55.8	Both	No
Nandy 2010/2 [37]	IGT	Healthy	VEGF-A	Plasma	America	Government/university grant	15	11	55.8	Both	No
Ozturk 2009 [38]	T2DM	Healthy	VEGF-A	Serum	Turkey	No information	31	28	63.9	Both	No
Ruszkowska-Ciastek 2014 [39]	T2DM	Healthy	VEGF-A	Serum	Poland	University grant	31	30	63.58	Both	No
Schlingemann 2013 [40]	T1DM DR- DN-	Healthy	VEGF-A	Plasma	Netherlands	No information	21	21	32	Both	No
Seckin 2006 [41]	T1DM (HbA1c > 8%)	T1DM (HbA1c ≤ 8%)	VEGF-A	Serum	Turkey	No information	70	30	9.97	Both	No
Siervo 2010 [42]	Metabolic syndrome	Healthy	VEGF-A, PIGF	Plasma	UK and Italy	Government grant	160	840	58.8	Both	Yes
Siervo 2012 [43]	Obese	Healthy	VEGF-A, PIGF	Plasma	Italy	Government grant	38	113	9.9	Both	Yes
Silha 2005 males [44]	Obese male	Healthy male	VEGF-A, VEGF-C, VEGF-D	Serum	Czech Republic, Canada	Government grant	4	24	46.3	Male	No
Silha 2005 females [44]	Obese female	Healthy female	VEGF-A, VEGF-C, VEGF-D	Serum	Czech Republic, Canada	Government grant	21	33	49.3	Female	No
Suguro 2008 [45]	T2DM	Healthy	VEGF-A	Plasma	Japan	Institutional/university grant	36	24	58	Both	Yes
Valabhji 2001 [46]	T1DM	Healthy	VEGF-A	Serum	UK	No information	41	50	39	Both	No
Wada 2010 [47]	Metabolic syndrome	Healthy	VEGF-A	Plasma	Japan	Government grant	43	229	47.2	Both	Yes
Wu 2017/1 [48]	T2DM	Healthy	VEGF-B	Plasma	China	No information	45	39	49	Both	No
Wu 2017/2 [48]	IGT	Healthy	VEGF-B	Plasma	China	No information	37	39	51	Both	No
Zorena 2010 [49]	T1DM	Healthy	VEGF-A	Serum	Poland	University grant	74	30	15	Both	No

*T1DM* type 1diabetes mellitus, *T2DM* type 2 diabetes mellitus, *IGT* impaired glucose tolerance, *DN* diabetic nephropathy, *HbA1c* glycated haemoglobin, *CVD* cardiovascular disease

### Quality of included studies

The quality of the included studies is presented in Table 2. Of the 32 included studies, 18 studies were rated as “poor”, 12 were rated “fair” and only two study was rated as “good” quality. Most of these studies were cross sectional, thus unable to establish temporality between exposure and outcome. This significantly limits the potential for any causal inference. In addition, most studies did not adjust for confounding variables in statistical analyses. This likely introduced bias into the reported associations. The sample sizes for many of these studies were small and lacked evidence of power calculations. Even when sample sizes were adequate, control participants were often drawn from populations sufficiently different from cases (e.g. hospital staff compared to patients) that any results from comparisons may be biased.

**Table 2:** Quality assessment of the included studies

Study	Q1	Q2	Q3	Q4	Q5	Q6	Q7	Q8	Q9	Q10	Q11	Q12	Q13	Q14	Rating
Doupis 2011 [50]	Yes	Yes	NR	Yes	No	No	No	Yes	Yes	No	Yes	CD	NA	Yes	Poor
Du 2016 [20]	Yes	Yes	NR	Yes	No	No	No	Yes	Yes	No	Yes	CD	NA	Yes	Poor
Erman 2016 [21]	Yes	Yes	NR	No	No	No	No	NA	Yes	No	Yes	CD	NA	No	Poor
Gomez-Ambrosi 2010 [9]	Yes	No	NR	Yes	No	No	No	NA	Yes	No	Yes	CD	NA	No	Poor
Guo 2014 [22]	No	Yes	NR	Yes	No	No	No	Yes	Yes	No	Yes	CD	NA	No	Poor
Hanefeld 2016 [23]	Yes	Yes	NR	CD	No	No	No	Yes	Yes	No	Yes	CD	NA	No	Poor
Jain 2013 [24]	Yes	Yes	NR	Yes	Yes	No	No	NA	Yes	No	Yes	CD	NA	Yes	Fair
Jesmin 2013 [25]	Yes	Yes	NR	No	No	Yes	Yes	NA	Yes	No	No	CD	Yes	No	Fair
Kakizawa 2004 [26]	Yes	Yes	NR	Yes	No	Yes	Yes	NA	Yes	No	Yes	CD	Yes	Yes	Good
Kubisz 2010 [27]	Yes	Yes	Yes	Yes	No	No	No	NA	Yes	No	Yes	CD	NA	Yes	Fair
Lim 2004 [28]	Yes	Yes	NR	Yes	No	No	No	No	Yes	No	Yes	CD	NA	No	Poor
Litvinova 2014 [29]	Yes	Yes	NR	Yes	No	Yes	No	Yes	Yes	No	Yes	CD	Yes	Yes	Fair
Loebig 2010 [30]	Yes	Yes	NR	CD	No	No	No	Yes	Yes	No	Yes	CD	NA	No	Poor
MacEneaney 2010 [31]	Yes	Yes	NR	Yes	No	No	No	Yes	Yes	No	Yes	CD	NA	No	Poor
Mahdy 2011 [32]	Yes	Yes	NR	Yes	No	NA	NA	NA	Yes	Yes	Yes	CD	Yes	Yes	Good
Marek 2010 [33]	Yes	Yes	NR	Yes	No	No	No	NA	Yes	No	Yes	CD	NA	Yes	Fair
Mirhafez 2015 [34]	Yes	Yes	NR	Yes	No	No	No	Yes	Yes	No	Yes	CD	NA	Yes	Fair
Mirhafez 2016 [35]	Yes	Yes	NR	No	No	No	No	Yes	Yes	No	Yes	CD	NA	No	Poor
Mysliwiec 2008 [36]	Yes	No	NR	No	No	No	No	No	Yes	No	Yes	CD	NA	No	Poor
Nandy 2010 [37]	Yes	Yes	NR	Yes	No	No	No	Yes	Yes	No	Yes	CD	NA	Yes	Fair
Ozturk 2009 [38]	Yes	Yes	NR	CD	No	No	No	NA	Yes	No	Yes	CD	NA	No	Poor
Ruszkowska-Ciastek 2014 [39]	Yes	Yes	NR	CD	No	No	No	Yes	Yes	No	Yes	CD	NA	No	Poor
Schlingemann 2013 [40]	Yes	Yes	NR	CD	No	No	No	Yes	Yes	No	Yes	CD	NA	No	Poor
Seckin 2006 [41]	Yes	Yes	NR	Yes	No	No	No	Yes	Yes	No	Yes	CD	NA	No	Poor
Siervo 2010 [42]	Yes	Yes	Yes	Yes	No	No	No	NA	Yes	No	Yes	CD	NA	Yes	Fair
Siervo 2012 [43]	Yes	Yes	Yes	Yes	No	No	No	NA	Yes	No	Yes	CD	NA	Yes	Fair
Silha 2005 [44]	Yes	Yes	NR	No	No	No	No	Yes	Yes	No	Yes	CD	NA	Yes	Fair
Suguro 2008 [45]	Yes	Yes	NR	Yes	No	Yes	No	NA	Yes	No	Yes	CD	Yes	No	Poor
Valabhji 2001 [46]	Yes	Yes	NR	No	No	No	No	NA	Yes	No	Yes	CD	NA	No	Poor
Wada 2010 [47]	Yes	Yes	NR	Yes	No	No	No	NA	Yes	No	Yes	CD	NA	Yes	Fair
Wu 2017 [48]	Yes	Yes	NR	Yes	No	Yes	No	No	Yes	No	Yes	CD	NA	Yes	Fair
Zorena 2010 [49]	Yes	No	NR	No	No	No	No	Yes	Yes	No	Yes	CD	NA	No	Poor

*CD* cannot determine, *NA* not applicable, *NR* not reported

Q1. Was the research question or objective in this paper clearly stated?, Q2. Was the study population clearly specified and defined?, Q3. Was the participation rate of eligible persons at least 50%?, Q4. Were all the subjects selected or recruited from the same or similar populations (including the same time period)? Were inclusion and exclusion criteria for being in the study prespecified and applied uniformly to all participants?, Q5. Was a sample size justification, power description, or variance and effect estimates provided?, Q6. For the analyses in this paper, were the exposure(s) of interest measured prior to the outcome(s) being measured?, Q7. Was the timeframe sufficient so that one could reasonably expect to see an association between exposure and outcome if it existed?, Q8. For exposures that can vary in amount or level, did the study examine different levels of the exposure as related to the outcome (e.g., categories of exposure, or exposure measured as continuous variable)?, Q9. Were the exposure measures (independent variables) clearly defined, valid, reliable, and implemented consistently across all study participants?, Q10. Was the exposure(s) assessed more than once over time?, Q11. Were the outcome measures (dependent variables) clearly defined, valid, reliable, and implemented consistently across all study participants?, Q12. Were the outcome assessors blinded to the exposure status of participants?, Q13. Was loss to follow-up after baseline 20% or less?, Q14. Were key potential confounding variables measured and adjusted statistically for their impact on the relationship between exposure(s) and outcome(s)?

### Association of VEGFs with metabolic syndrome or its components—meta-analysis

**Fig. 2:**
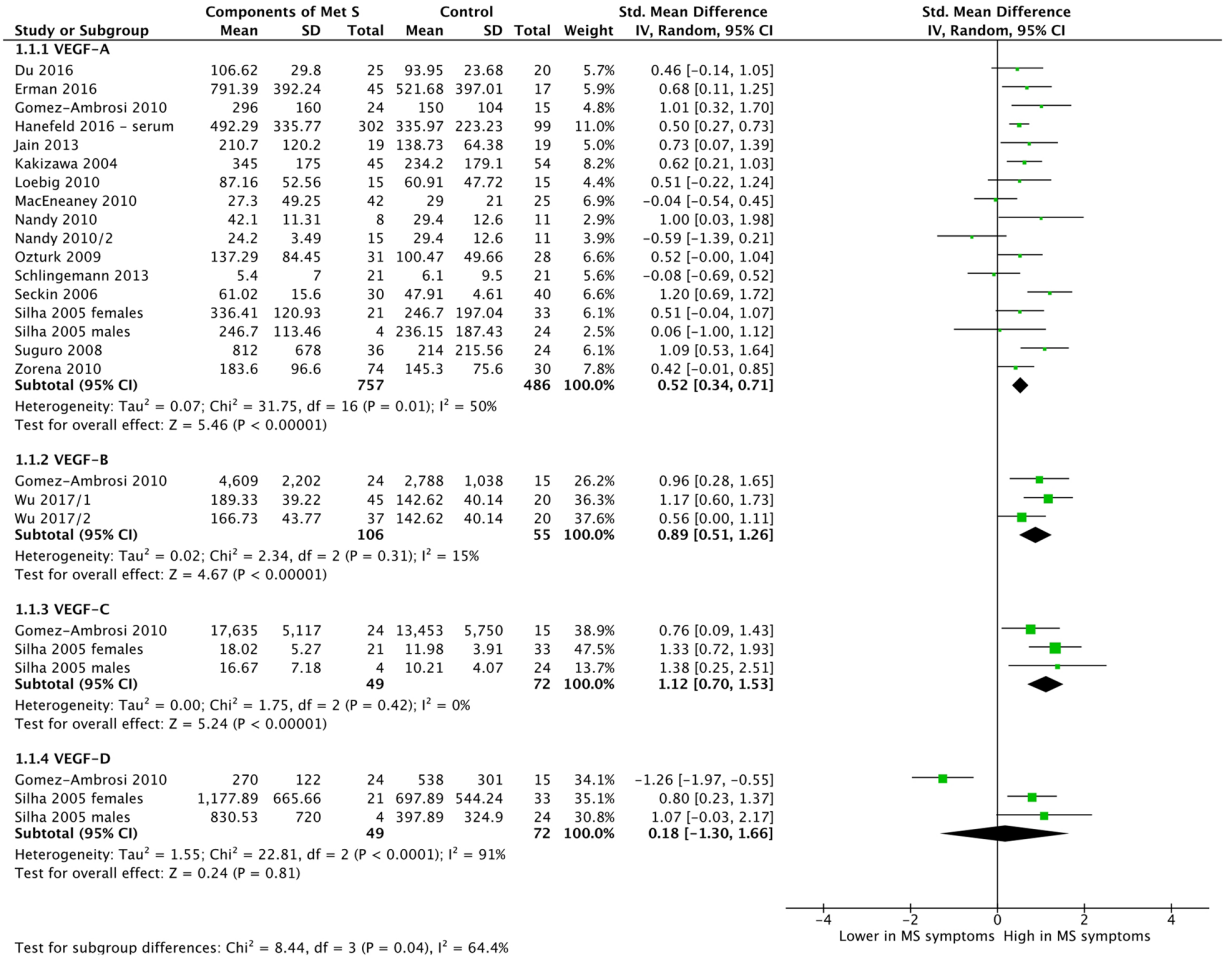
Subgroup meta-analysis of VEGF expression by VEGF protein

We undertook a subgroup meta-analysis of VEGFs expression in people with and without metabolic syndrome or its components by VEGF protein (Fig. 2). Most studies measured VEGF-A concentrations, with only two studies each reporting on VEGF-B, VEGF-C or VEGF-D. In all cases except VEGF-D, the expression of the VEGF family was higher in people with metabolic syndrome or components of the metabolic syndrome. Expression of VEGF-A was significantly higher (SMD = 0.52; 95% CI 0.34, 0.71; p < 0.0001) in people with metabolic syndrome or components thereof. This equates to an increase in VEGFs expression of over 50 pg/mL. The two studies (three study arms) reporting on VEGF-B expression, as well as the two studies (three study arms) reporting on VEGF-C, were associated with significantly higher VEGF expression (p < 0.0001, p < 0.0001, respectively). Heterogeneity as measured by *I*^*2*^ varied from 0% (VEGF-C) to 91% (VEGF-D). The latter was driven entirely by Gomez-Ambrosi [9].

**Fig. 3:**
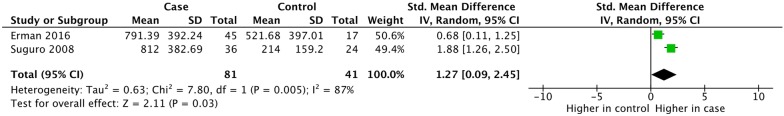
Meta-analysis of VEGF-A expression in metabolic syndrome. Metabolic syndrome was defined as at least three of: obesity, hypertension, low HDL, hyperglycemia, hypertriglyceridemia or high LDL

To investigate differences in VEGFs expression based on different metabolic syndrome components, we undertook a meta-analysis of studies of people with at least three of the five components of the metabolic syndrome, or where the study authors stated that the cohort had metabolic syndrome (Fig. 3). Only two studies could be included in the meta-analysis. However, these two studies both demonstrated a significant increase in VEGF-A expression, and together were statistically significant (SMD = 1.27; 95% CI 0.09, 2.45; p = 0.03). This equates to a mean difference in VEGF-A expression of approximately 150 pg/ml. Heterogeneity as measured by *I*^*2*^ was high (87%).

**Fig. 4:**
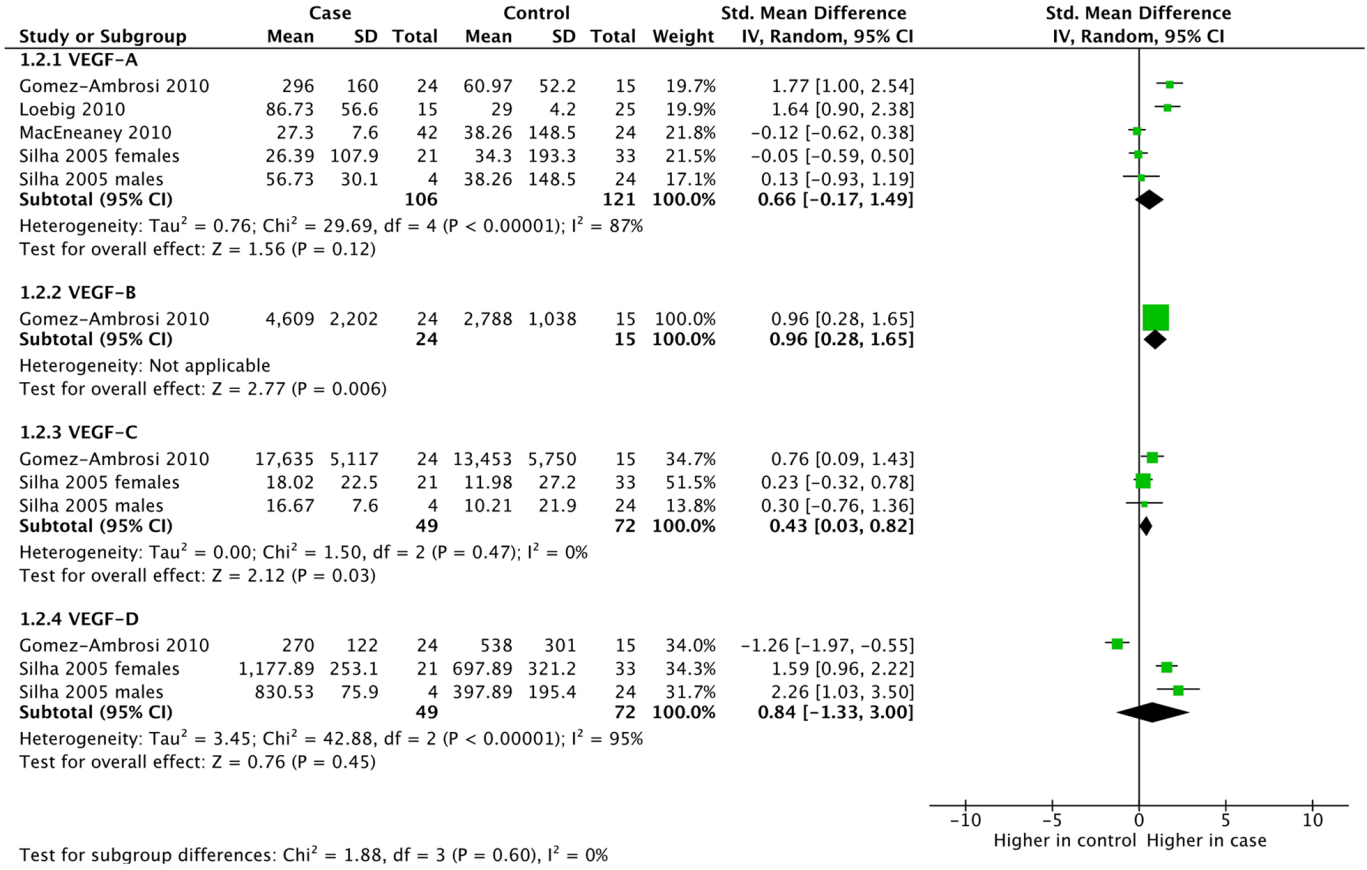
Subgroup meta-analysis of VEGF expression in obesity by VEGF protein. Obesity was defined as a mean BMI greater than 30

Several studies measured VEGF concentrations in people with or without obesity. We performed a subgroup meta-analysis of studies including people with obesity (Fig. 4). This meta-analysis included 12 study arms from four individual studies. Neither VEGF-A nor VEGF-D expression was increased in people with obesity (SMD = 0.66; 95% CI − 0.17, 1.49; p = 0.12) and (SMD = 0.84; 95% CI − 1.33, 3.00; p = 0.45), respectively. Whereas, the studies in people with obesity that measured VEGF-B (p = 0.006) and VEGF-C (p = 0.03) showed statistically significantly higher VEGF-B or C protein expression in people with obesity. Heterogeneity as measured by *I*^*2*^ varied from 0% (VEGF-C) to 95% (VEGF-D). The latter was driven entirely by Gomez-Ambrosi 2010 [9].

**Fig. 5:**
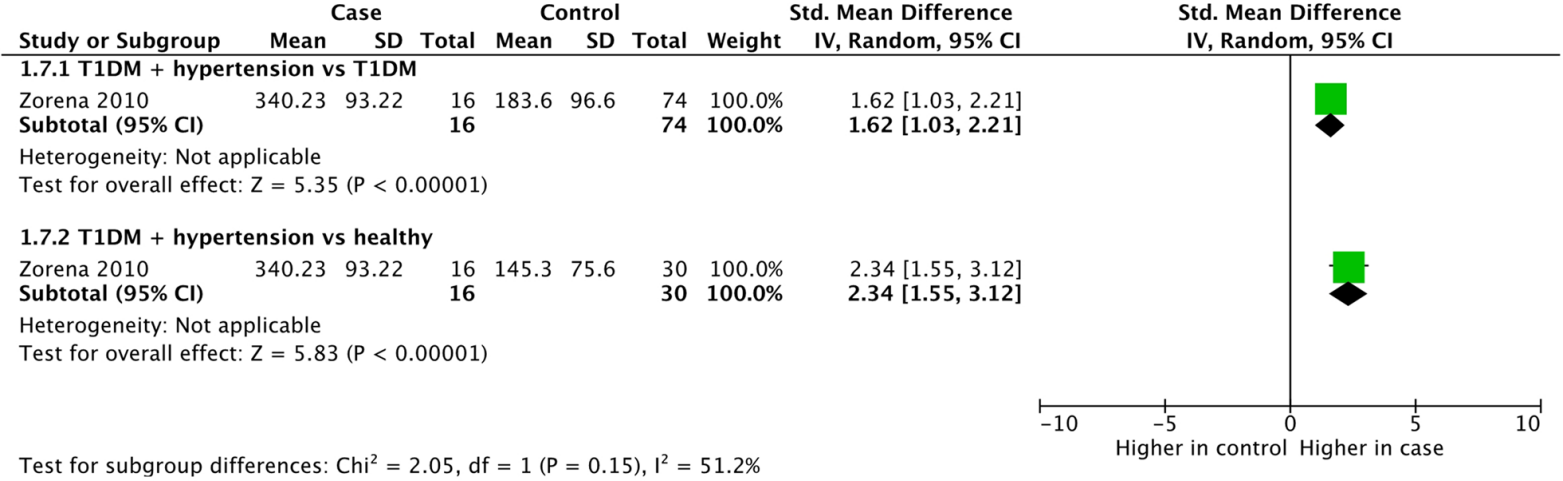
Meta-analysis of VEGF-A expression in hypertension. Hypertension was defined as a systolic blood pressure over 130 mm Hg, and/or a diastolic blood pressure over 85 mm Hg

Hypertension is an important component of metabolic syndrome. One study looked at the effect of hypertension in children with type 1 diabetes (Fig. 5). Compared with healthy children, VEGF concentrations were much higher in children with type 1 diabetes and hypertension (SMD = 2.34, 95% CI 1.55, 3.12, p < 0.0001). Similarly, hypertension was strongly associated with increased VEGF concentrations even compared with children with type 1 diabetes and normal blood pressure (SMD = 1.62, 95% CI 1.03, 2.21, p < 0.0001). We intended to analyse the correlation of hypertension with different VEGF proteins, but due to a lack of studies we weren’t able to perform this analysis.

**Fig. 6:**
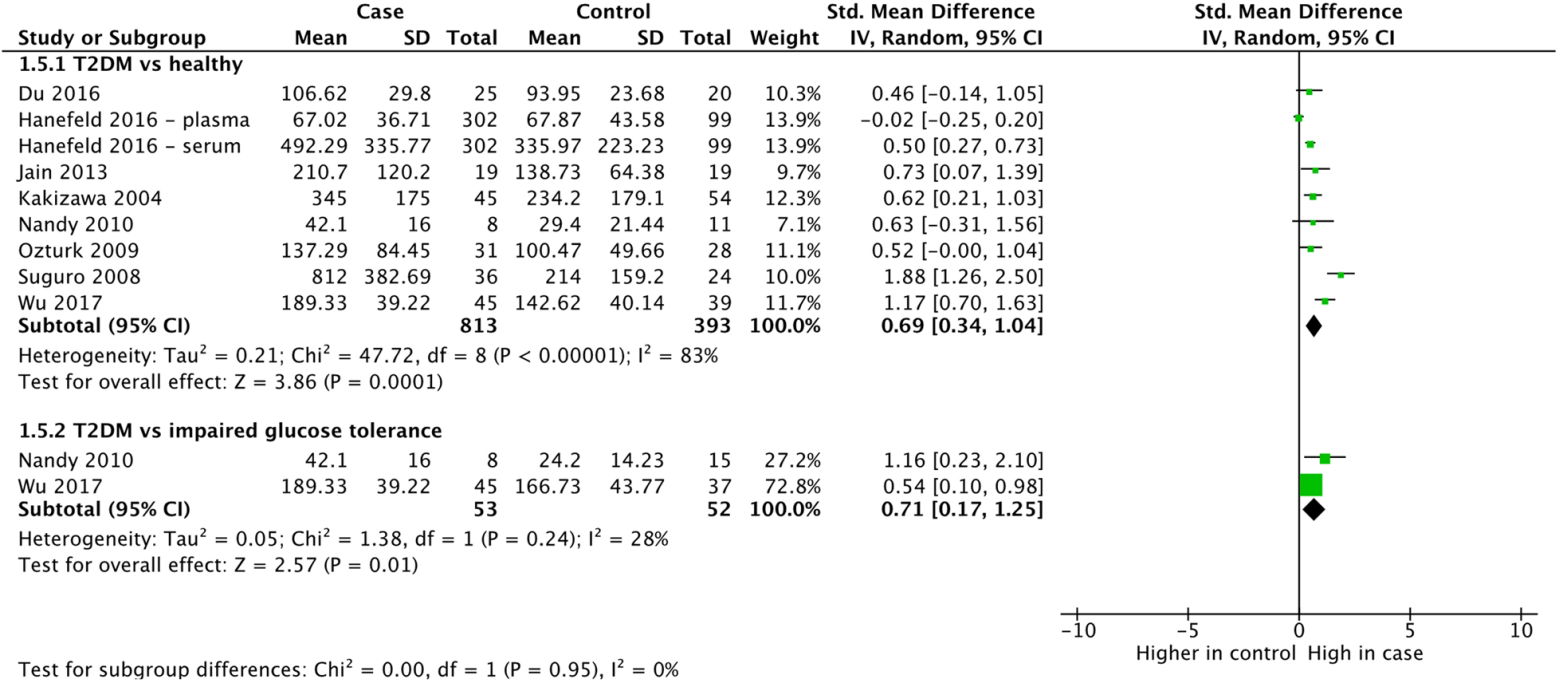
Subgroup meta-analysis of VEGF-A or VEGF-B expression in hyperglycemia by VEGF protein. Hyperglycemia was defined as a fasting blood glucose concentration over 100 mg/dl (> 5.6 mmol/L). VEGF-B was analyzed in Wu 2017. All other studies analyzed VEGF-A

Hyperglycemia, either as part of diabetes or impaired glucose tolerance (pre-diabetes), is another important component of metabolic syndrome. We included 11 study arms from 8 individual studies (Fig. 6), all of which measured VEGF-A. The analysis of studies comparing those with type 2 diabetes and healthy controls demonstrated a strong association between hyperglycemia and increased VEGF expression (SMD = 0.69, 95% CI 0.34, 1.04; p = 0.0001), although heterogeneity was high (*I*^*2*^ = 83%). When comparing people with type 2 diabetes and people with impaired glucose tolerance, a significant increase in VEGF was still observed (SMD = 0.71, 95% CI 0.17, 1.25, p = 0.01). We intended to analyse the correlation of hyperglycemia with different VEGF proteins, but due to a lack of this studies we weren’t able to perform this analysis.

**Fig. 7:**
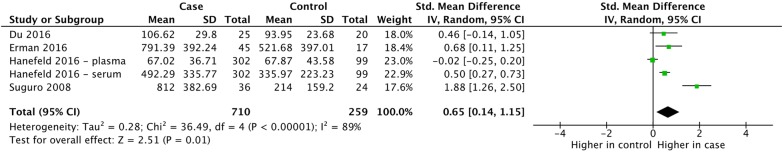
Meta-analysis of VEGF-A expression in hypertriglyceridemia. Hypertriglyceridemia was defined as fasting blood triglycerides over 150 mg/dl (> 1.7 mmol/L)

There were no studies specifically targeting patients with hypertriglyceridemia. However, we took the four studies in people with high triglycerides in addition to other conditions and undertook a meta-analysis (Fig. 7). The meta-analysis of the five included study arms was associated with a significant increase in VEGF-A expression (SMD = 0.65, 95% CI 0.14, 1.15, p = 0.01). Heterogeneity was high (*I*^*2*^ = 89%).

**Fig. 8:**
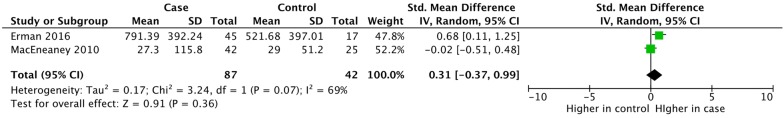
Meta-analysis of VEGF-A expression in high LDL-C. High LDL-C was defined as fasting LDL-D over 131 mg/dl (> 3.4 mmol/L)

There were no studies specifically targeting patients with raised LDL-cholesterol. We therefore took the two studies in people with high LDL-cholesterol in addition to other conditions and undertook a meta-analysis (Fig. 8). The meta-analysis of these studies did not show an association between high LDL-C and higher VEGF expression (SMD = 0.31, 95% CI − 0.37, 0.99, p = 0.36). Heterogeneity was moderate (*I*^*2*^ = 69%). There were no studies in people with low HDL-cholesterol.

**Fig. 9:**
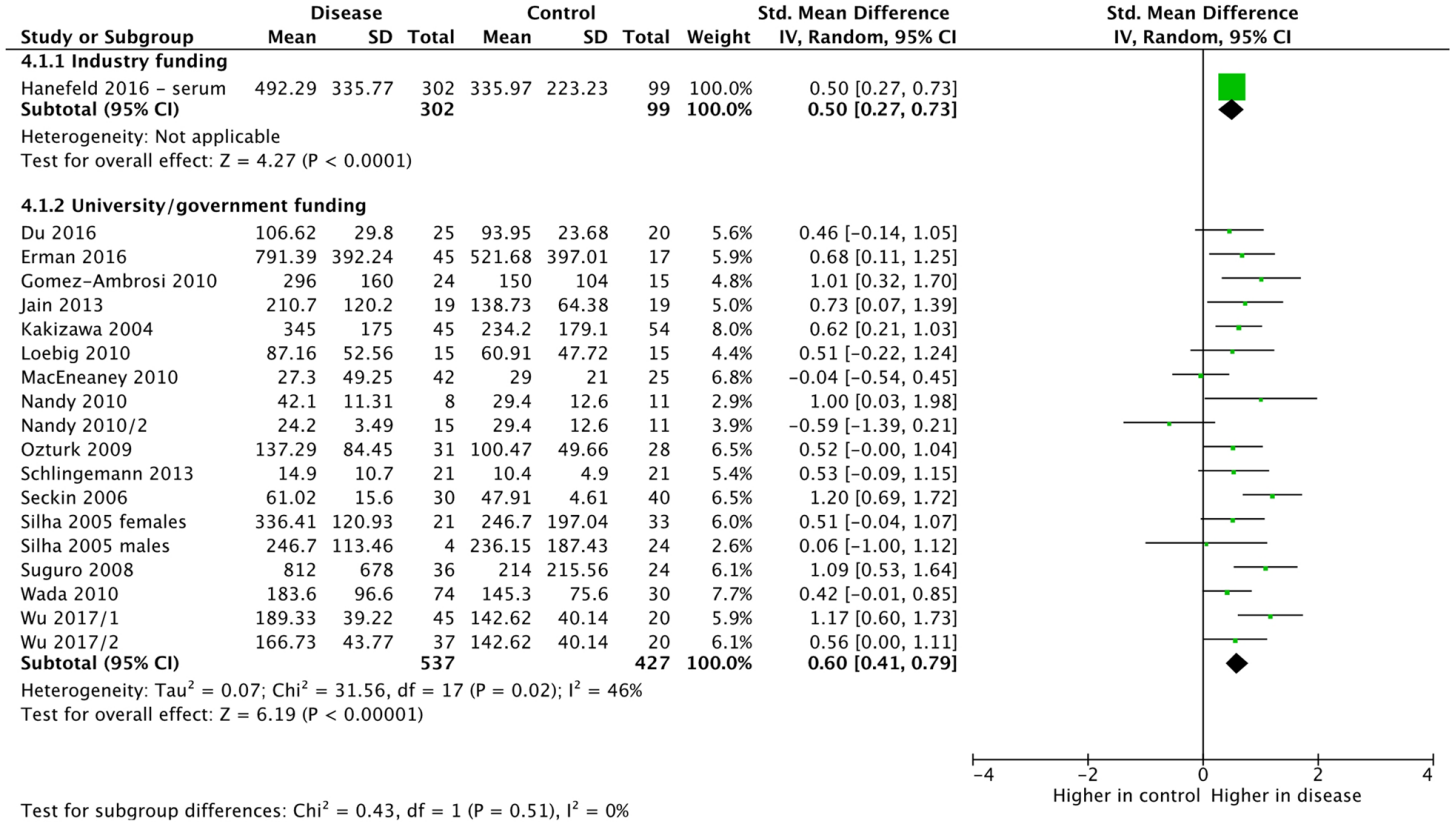
Subgroup meta-analysis of VEGF-A or VEGF-B expression by funding source. VEGF-B was analyzed in Wu 2017. All other studies analyzed VEGF-A

**Fig. 10:**
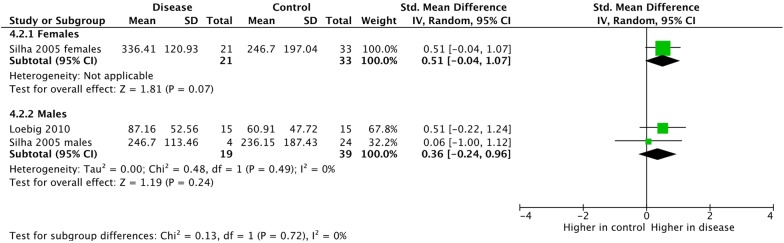
Subgroup meta-analysis of VEGF-A expression by gender

**Fig. 11:**
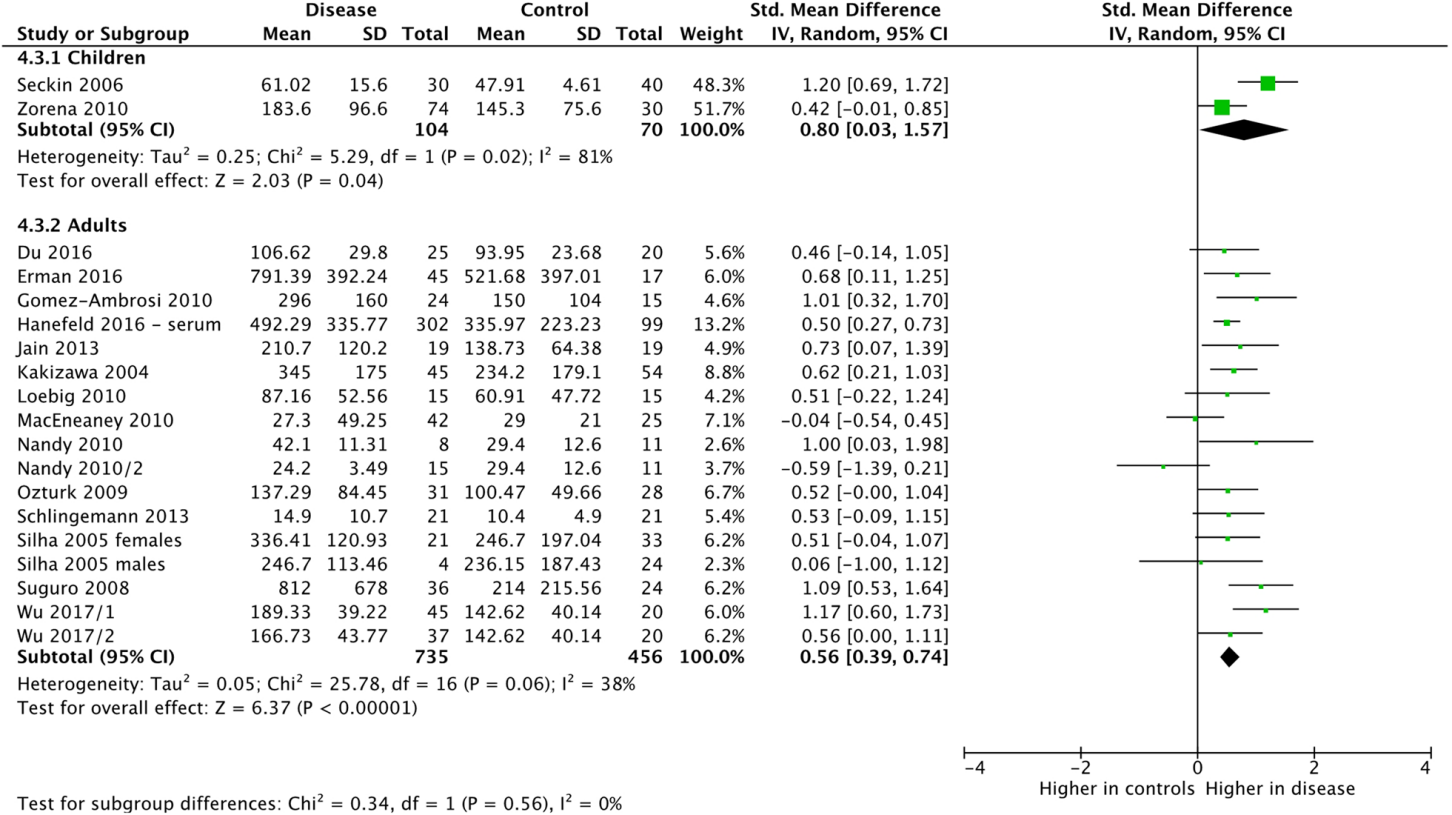
Subgroup meta-analysis of VEGF-A or VEGF-B expression by age. VEGF-B was analyzed in Wu 2017. All other studies analyzed VEGF-A

As stated earlier, we intended to undertake subgroup or sensitivity analysis based on concomitant medication, funding source, age of participants, co-morbidities of participants and gender of participants. Due to the paucity of data, we could only undertake subgroup analyses by funding source (Fig. 9), gender (Fig. 10) and age (Fig. 11). There were no significant differences between any subgroups. The differences in plasma compared with serum VEGF led us to undertake a post hoc sensitivity analysis of VEGF-A. Removing the studies that measured plasma VEGF-A from the overall analysis (thus including serum VEGF-A only) led to a slight increase in the SMD (0.63, 95% CI 0.45, 0.80, p < 0.0001) and a reduction of the heterogeneity as measured by *I*^*2*^ from 50 to 11%. The studies measuring plasma VEGF-A concentrations resulted in a smaller standardized mean difference that was also statistically significant (SMD = 0.44, 95% CI 0.06, 0.81, p = 0.02).

### Association of VEGFs with metabolic syndrome or its components—qualitative results

VEGF in some studies was not normally distributed. In these cases, the data were reported as medians and interquartile ranges. Although it is possible to approximate a standard deviation from such data, it is not recommended [51]. Nevertheless, such data can provide valuable additional support to meta-analyzed data. Table 3 contains studies that could not be included in the meta-analysis. The ratio between the median VEGF concentration in the metabolic syndrome cohort and the control cohort was calculated. In the majority of studies, the ratio was greater than 1 (range 0.47–2.72), suggesting that VEGF concentrations were higher in people with component of metabolic syndrome or the metabolic syndrome itself.

**Table 3:** Median VEGF superfamily expression in people with and without metabolic syndrome or its components

	Gene	Disease group (pg/ml)	Control group (pg/ml)	Ratio
Metabolic syndrome				
Doupis 2011/2 [50]	VEGF-A	139	88	1.58
Jesmin 2013 [25]	VEGF-A	483.93	386.88	1.25
Lim 2004/1 [28]	VEGF-A	200	90	2.22
Lim 2004/2 [28]	VEGF-A	180	90	2.00
Litvinova 2014 [29]	VEGF-A	180	172	1.05
Mirhafez 2015 [34]	VEGF-A	38.55	82.18	0.47
Mirhafez 2016 [35]	VEGF-A	85.5	81.1	1.05
Siervo 2010 [42]	VEGF-A	431.8	350.6	1.23
Siervo 2010 [42]	PIGF	13.5	11.5	1.17
Wada 2010 [47]	VEGF-A	332.3	268.8	1.24
Hyperglyecmia				
Doupis 2011/1 [50]	VEGF-A	165	88	1.88
Doupis 2011/2 [50]	VEGF-A	139	88	1.58
Guo 2014 [22]	VEGF-A	269.41	211.36	1.27
Jesmin 2013 [25]	VEGF-A	483.93	386.88	1.25
Kubisz 2010 [27]	VEGF-A	338.5	182	1.86
Lim 2004/1 [28]	VEGF-A	200	90	2.22
Lim 2004/2 [28]	VEGF-A	180	90	2.00
Mahdy 2011 [32]	VEGF-A	16.25	6.35	2.56
Marek 2010 [33]	VEGF-A	117.43	113.03	1.04
Mirhafez 2015 [34]	VEGF-A	38.55	82.18	0.47
Mirhafez 2016 [35]	VEGF-A	85.5	81.1	1.05
Mysliwiec 2008 [36]	VEGF-A	172	93.66	1.84
Ruszkowska-Ciastek 2014 [39]	VEGF-A	11.15	12.13	0.92
Obesity				
Doupis 2011/1 [50]	VEGF-A	139	88	1.58
Doupis 2011/2 [50]	VEGF-A	239	88	2.72
Litvinova 2014 [29]	VEGF-A	180	172	1.05
Mirhafez 2015 [34]	VEGF-A	38.55	82.18	0.47
Mirhafez 2016 [35]	VEGF-A	85.5	81.1	1.05
Ruszkowska-Ciastek 2014 [39]	VEGF-A	11.15	12.13	0.92
Siervo 2010 [42]	VEGF-A	431.8	350.6	1.23
Siervo 2010 [42]	PIGF	13.5	11.5	1.17
Siervo 2012 [43]	VEGF-A	341	264	1.29
Siervo 2012 [43]	PIGF	14	12.2	1.15
Hypertension				
Doupis 2011/1 [50]	VEGF-A	139	88	1.58
Doupis 2011/2 [50]	VEGF-A	239	88	2.72
Guo 2014 [50]	VEGF-A	269.41	211.36	1.27
Lim 2004/1 [28]	VEGF-A	200	90	2.22
Lim 2004/2 [28]	VEGF-A	180	90	2.00
Mahdy 2011 [32]	VEGF-A	16.25	6.35	2.56
Mirhafez 2015 [34]	VEGF-A	38.55	82.18	0.47
Ruszkowska-Ciastek 2014 [39]	VEGF-A	11.15	12.13	0.92
Siervo 2010 [42]	VEGF-A	431.8	350.6	1.23
Siervo 2010 [42]	PIGF	13.5	11.5	1.17
Valabhji 2001 [46]	VEGF-A	217	137	1.58
Wada 2010 [47]	VEGF-A	332.3	268.8	1.24
Low HDL				
Jesmin 2013 [25]	VEGF-A	483.93	386.88	1.25
Mirhafez 2016 [35]	VEGF-A	85.5	81.1	1.05
High triglycerides				
Doupis 2011/2 [50]	VEGF-A	139	88	1.58
Jesmin 2013 [25]	VEGF-A	483.93	386.88	1.25
Lim 2004/1 [28]	VEGF-A	200	90	2.22
Lim 2004/2 [28]	VEGF-A	180	90	2.00
Mirhafez 2016 [35]	VEGF-A	85.5	81.1	1.05
Siervo 2010 [42]	VEGF-A	431.8	350.6	1.23
Siervo 2010 [42]	PIGF	13.5	11.5	1.17
Wada 2010 [47]	VEGF-A	332.3	268.8	1.24
High LDL				
Lim 2004/1 [28]	VEGF-A	200	90	2.22
Lim 2004/2 [28]	VEGF-A	180	90	2.00
Wada 2010 [47]	VEGF-A	332.3	268.8	1.24

### Publication bias

**Fig. 12:**
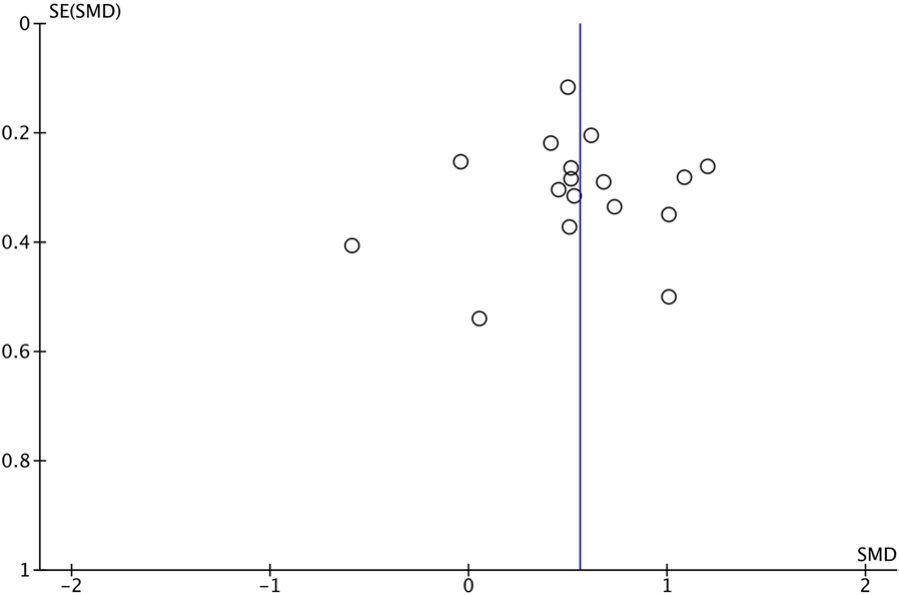
Funnel plot analysis of all studies included in the meta-analysis. Standardized mean difference (SMD) in VEGF-A expression was plotted against standard error of the SMD

In order to determine if publication bias exists, we undertook a funnel plot analysis (Fig. 12). Overall, the plot appears to be largely symmetrical, although it is possible that some low-quality studies showing increased VEGF expression were missing. We undertook the random effects version Egger’s regression test [18] to assess funnel plot asymmetry based on standard error. The test did not indicate any evidence of asymmetry (p = 0.67).

## Discussion

Both quantitative and qualitative data from our study suggest that VEGF-A is overexpressed in the presence of metabolic syndrome. Further, our subgroup analyses show that with increasing disease, the concentration of VEGF proteins increases. That is, for example, the difference in VEGF-A expression in healthy children compared with children with type 1 diabetes and hypertension is greater than the difference between children with type 1 diabetes and those with type 1 diabetes and hypertension. This finding is not unexpected, as VEGF-A is known for its proangiogenic effects.

Interestingly, the subgroup analysis identified a significant association between increased levels of VEGF-A and hyperglycemia, but not obesity. The latter represents a very surprising finding, as angiogenesis is a process involved in adipose tissue expansion that takes place in obesity [5, 52]. Nonetheless, given the fact that the cohort of obese patients included in the quantitative analysis mostly did not suffer from metabolic complications, the authors speculate that, in conditions of preserved insulin sensitivity, obesity is not a stimulus strong or effective enough to stimulate VEGF-A expression. It is known that hypoxia is one the strongest stimuli for VEGF-A expression, thus, in the absence of IR-induced vasoconstriction and consequent hypoxia, VEGF-A may remain within its physiological limits However, although on a small cohort of patients, our study showed significantly increased levels of the angiogenic and lymphangiogenic factors VEGF-B and VEGF-C in obese patients, which may suggest that the heterogeneity among studies that addressed VEGF-A expression may have influenced the obtained results. Our finding of a correlation between VEGF-B and obesity corresponds to the findings observed by Robciuc et al. They observed that increased VEGF-B expression in the adipose tissue of a Vegfb transgenic mouse model increased capillary density, tissue perfusion, and insulin supply by the binding of VEGF-B to VEGFR1, which then activates the VEGF-A/VEGFR2 pathway [11]. On the other hand, VEGF-B is a key trans-endothelial FA regulator and as such, it would be expected for its expression to decrease in response to high amounts of circulating FAs [53, 54]. While it is possible that increased VEGF-B signaling is responsible for the pathological lipid accumulation in obese patients, further research is needed to elucidate this hypothesis.

The mechanism of the interaction between VEGF-A and LDL-C is still largely unknown. Our study did not identify a significant increase of VEGF-A levels in patients with high LDL-C levels, and this finding may be a result of the fact that the participants had differences in other comorbidities, or that the trans-endothelial transport of LDL-C is not influenced and does not depend on the presence of VEGF-A in the cell culture medium, as recently shown by Velagapudi et al. [55]. This may explain the earlier findings of Sandhofer et al. who failed to detect any association between VEGF-A levels and carotid atherosclerosis [56]. Further studies are needed to elucidate the exact behavior of VEGF-A and its correlation with LDL-C.

Mechanistically, it appears that the propensity towards higher concentrations of VEGF-A is, at least in part, genetically determined. Recently, Ghazizadeh et al. examined the association of a mutation in the *VEGFA* gene. They found association with an A-to-G mutation in the rs10738760 SNP and metabolic syndrome [57]. Interestingly, it appears that increased VEGF expression could both be a response to and a cause of increasing disease. However, this study was retrospective, and longitudinal studies will be required to elucidate the role of the VEGFs as a causative agent of disease.

Despite the somewhat heterogeneous nature of the available data, our study strongly suggests that expression of VEGFs differs among patients with metabolic syndrome. Although the regulation of the expression of VEGF proteins in preserved and impaired insulin signalling conditions remains largely unknown, our data synthesize the available evidence for the first time and provide a numerical estimate of the above-mentioned differences. The data from our meta-analyses are further supported by qualitative data, specifically by the ratio between the median VEGF concentration in the metabolic syndrome cohorts and the control cohorts. This ratio was greater than 1 in most of the studies (range 0.47–2.72), suggesting that VEGF concentrations were higher in people with a component of metabolic syndrome or the metabolic syndrome itself. This effect was particularly pronounced in the case of hypertriglyceridemia, with 7 out 8 included cohorts presenting ratios greater than 1 in favor of the high triglycerides cohort. The ratio between VEGF concentrations in the high LDL cohort and the control cohort was greater than 1 in all of the included studies (3/3) with two-fold increased VEGF concentrations in 2 out 3 studies. It has been shown that both VEGF-A and VEGF-B have the potential to increase vessel perfusion in obese adipose tissue, while VEGF-C and VEGF-D have lymphangiogenic properties [5, 11, 12, 46, 52]. In a pro-inflammatory milieu of excess FAs and/or glucose as found in obese and IR patients, the properties of these proteins are vital for two reasons: (1) enhanced vascularity increases the availability of insulin to target organs and improves insulin sensitivity, and (2) insulin-induced capillary recruitment and increased blood flow facilitate glucose uptake in target organs [58]. This especially refers to VEGF-A, which has been found to reduce metabolic complications caused by a high-fat diet and in the metabolic syndrome, through enhanced vascularity, thermogenesis and a decrease in inflammation [5, 52].

Interestingly, the expression of VEGFs in plasma and serum differs. When Hanefeld et al. examined the association of serum and plasma VEGF-A with type 2 diabetes [23], they found an order of magnitude difference in the concentration of VEGF-A in serum vs plasma. The serum concentrations of VEGF-A were 336 and 492 ng/l in control and type 2 diabetes patients, respectively. The difference between the groups was highly significant (p < 0.001). In contrast, the plasma VEGF-A concentrations in control and type 2 diabetes patients were 67.87 and 67.02 ng/l (p = 0.66) [23]. The authors suggested that the difference between serum and plasma VEGF-A concentrations is the result of VEGF-A accumulation in activated platelets [23]. We tested this hypothesis with a sensitivity analysis. Indeed, our results showed that removal of studies using plasma VEGF concentrations increased the SMD between patient and control groups.

### Strengths and limitations

We acknowledge the limitations in our study. Firstly, there was significant heterogeneity among the 16 studies on VEGF-A (*I*^*2*^= 50%) and 2 studies on VEGF-D (*I*^*2*^= 91%), included in this meta-analysis. This suggests that there were differences between studies that cannot be accounted for by chance; for example, co-morbidities, medication use, age or others. Unfortunately the lack of studies prevented us from investigating this heterogeneity more thoroughly. Secondly, the study populations of the included studies were small in number and this may restrict the generalization of our findings. However, these results should be regarded as preliminary. The strength of this meta-analysis lies in the fact that it is the first to explore the correlation between VEGFs overexpression and metabolic syndrome or its components, and in the significant number of studies reporting on the expression of VEGF-A.

## Conclusion

Overall the findings of this study demonstrate the strong association of increase in VEGFs expression with metabolic syndrome as well as its components. Our data strongly suggest overexpression of VEGF-A in patients with metabolic syndrome, hyperglycemia, hypertriglyceridemia and hypertension, but not obesity and high LDL. Preliminary data on the lymphangiogenic factors VEGF-C and VEGF-D suggest there are significantly increased and significantly decreased levels of these two proteins in metabolic syndrome or its components, respectively. However, further clinical studies on the association for the components of metabolic syndrome and VEGFs with the potential to adjust for confounding factors are encouraged.
